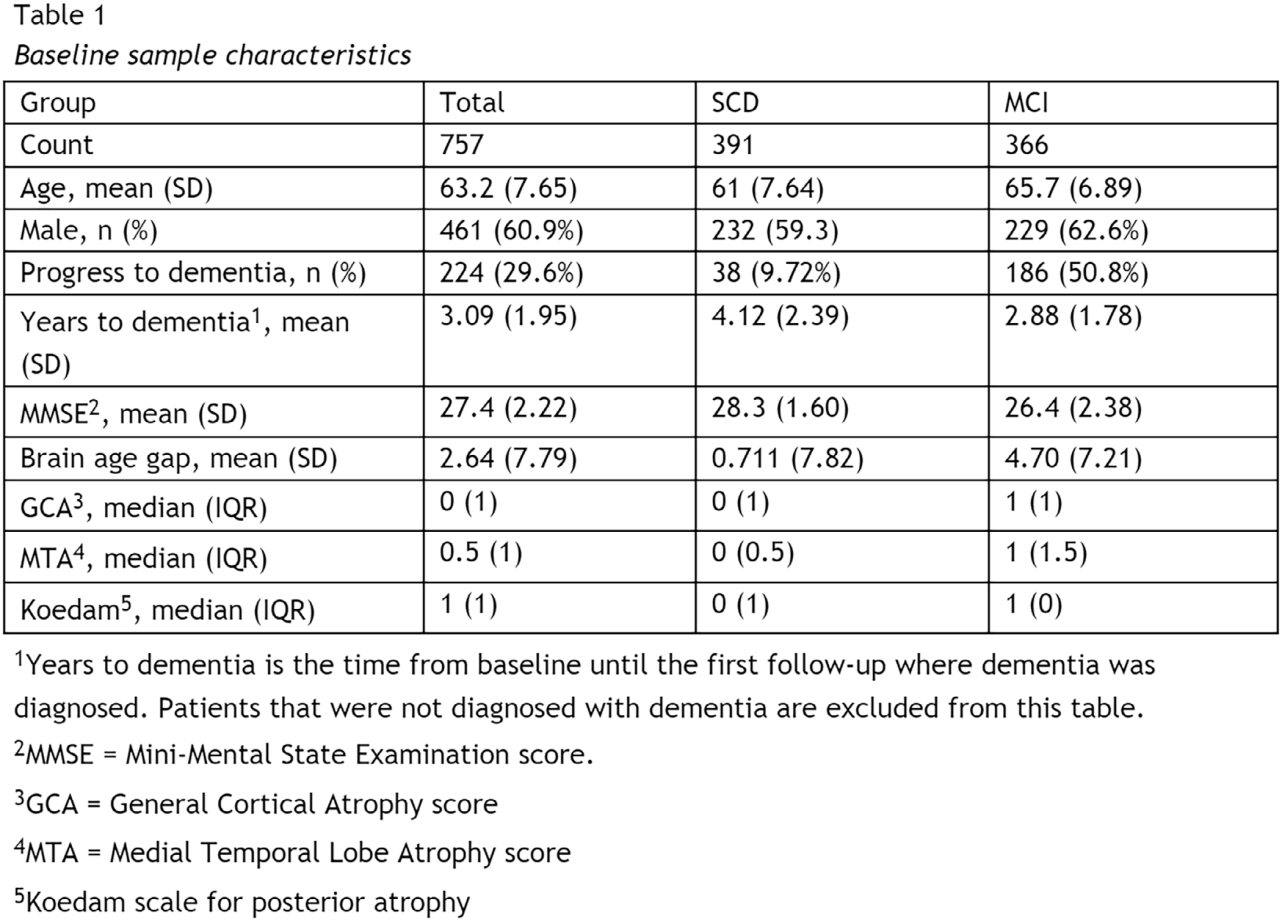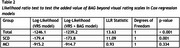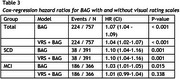# Brain Age Gap Predicts Progression to Dementia in SCD and MCI

**DOI:** 10.1002/alz70856_103050

**Published:** 2025-12-25

**Authors:** Stefan de Vries, Katalin Farkas, Mara ten Kate, Hanneke F.M. Rhodius‐Meester, Calvin Trieu, Alle Meije Wink, Jyrki Lötjönen, Wiesje M. van der Flier, Frederik Barkhof, Bas Jasperse

**Affiliations:** ^1^ Department of Radiology & Nuclear Medicine, Vrije Universiteit Amsterdam, Amsterdam UMC, location VUmc, Amsterdam, Netherlands; ^2^ Department of Geriatric Medicine, The Memory Clinic, Oslo University Hospital, Oslo, Norway; ^3^ Amsterdam Neuroscience, Neurodegeneration, Amsterdam, Netherlands; ^4^ Alzheimer Center Amsterdam, Neurology, Vrije Universiteit Amsterdam, Amsterdam UMC location VUmc, Amsterdam, Netherlands; ^5^ Combinostics Oy, Tampere, Finland; ^6^ Epidemiology and Biostatistics, Vrije Universiteit Amsterdam, Amsterdam UMC location VUmc, Amsterdam, Netherlands; ^7^ University College London (UCL), London, United Kingdom

## Abstract

**Background:**

Early identification of individuals at risk for dementia is crucial for timely diagnosis and intervention. Visual rating scales (VRS) for atrophy on MRI are established tools in clinical practice for assessing dementia‐related brain changes and have predictive value for progression to dementia in mild cognitive impairment (MCI). However, the coarseness of VRS limits predictive value earlier in the disease. Alternatively, brain age gap (BAG), the difference between chronological and age estimated from MRI using AI, shows promise in detecting dementia‐related brain changes. In this study we assess whether BAG can predict progression to dementia in those with subjective cognitive decline (SCD) and MCI.

**Method:**

We included subjects from the Amsterdam Dementia Cohort with a diagnosis of SCD (*n* = 391) or MCI (*n* = 366), baseline MRI, and at least 2 years of prospective clinical follow‐up (Table 1). T1 MR images were processed with SPM12 followed by BrainageR to derive BAG, which were adjusted for age, sex, and MRI scanner. Cox regression was used to model the risk of clinical progression to dementia. These models included age, sex, MMSE, and BAG, and hazard ratios (HR) were calculated to assess predictive value of BAG. To assess the added value of BAG over traditional radiological assessment, we repeated the analysis adding VRS of medial temporal lobe atrophy, global atrophy and parietal atrophy to the model. Analyses were performed in the whole sample and stratified per disease stage (SCD/MCI).

**Result:**

224 subjects progressed to dementia (SCD=38 (9.72%), MCI=186 (50.8%)). On average, a dementia diagnosis was given 3.09 years (SD=1.95) after baseline. BAG predicted progression to dementia, with added value beyond VRS (Table 2). The predictive value of BAG was strongest in SCD, where VRS provided no added value. In MCI, the predictive value of BAG was lower and insignificant when VRS were included (Table 3).

**Conclusion:**

BAG is predictive of progression to dementia in SCD and MCI and has added value over VRS in SCD. This highlights the potential of BAG as a predictor of progression to dementia, especially in early disease stages, when traditional VRS are insensitive.